# Comparative Study on Microencapsulation of Lavender (*Lavandula angustifolia* Mill.) and Peppermint (*Mentha piperita* L.) Essential Oils via Spray-Drying Technique

**DOI:** 10.3390/molecules26247467

**Published:** 2021-12-09

**Authors:** Bissera Pilicheva, Yordanka Uzunova, Plamen Katsarov

**Affiliations:** 1 Department of Pharmaceutical Sciences, Faculty of Pharmacy, Medical University of Plovdiv, 4002 Plovdiv, Bulgaria; bisera.pilicheva@mu-plovdiv.bg; 2 Research Institute at Medical University of Plovdiv (RIMU), 4002 Plovdiv, Bulgaria; yordanka.uzunova@mu-plovdiv.bg; 3 Department of Bioorganic Chemistry, Faculty of Pharmacy, Medical University of Plovdiv, 4002 Plovdiv, Bulgaria

**Keywords:** spray-drying, lavender oil, peppermint oil, microencapsulation, Arabic gum, maltodextrin

## Abstract

Essential oils have been studied for various applications, including for therapeutic purposes. There is extensive literature regarding their properties; however, their low stability limits their application. Generally, the microencapsulation of essential oils allows enhanced stability and enables the potential incorporation in solid dosage forms. Lavender and peppermint oils were encapsulated in microparticles using a spray-drying technique under optimized conditions: 170 °C temperature, 35 m^3^/h aspiration volume flow, and 7.5 mL/min feed flow. Arabic gum and maltodextrin were used as coating polymers individually in varying concentrations from 5 to 20% (*w/v*) and in combination. The microparticles were studied for morphology, particle size, oil content, and flowability. The formulated powder particles showed a high yield of 71 to 84%, mean diameter 2.41 to 5.99 µm, and total oil content of up to 10.80%. The results showed that both the wall material type and concentration, as well as the type of essential oil, significantly affected the encapsulation process and the final particle characteristics. Our study has demonstrated that the encapsulation of lavender and peppermint oils in Arabic gum/maltodextrin microparticles by spray-drying represents a feasible approach for the conversion of liquids into solids regarding their further use in powder technology.

## 1. Introduction

Essential oils (EOs) are widely used as natural preservatives, fragrances, or flavors [1,2,3]. Recently, significant data concerning various therapeutic applications have been compiled, which entails the evaluation of their capacity for incorporation in various delivery systems. Lavender oil, which is generally obtained from a species of the family *Lamiaceae* (*Lavandula angustifolia*, L. *latifolia*, L. *stoechas,* and L. *intermedia*), has a variety of cosmetic and therapeutic applications in herbal medicine ranging from relaxation to treating parasitic infections, burns, and insect bites [4,5,6]. Recent evidence suggests that lavender oil may prove to be effective in the treatment of neurological disorders, such as anxiety, mood instability, convulsions, neurodegenerative disorders, etc. [7]. Peppermint oil, which is obtained from the leaves of *Mentha piperita* L., *Lamiaceae*, is a well-known and important phytoproduct widely used in traditional medicine for several thousand years. The chemical composition of peppermint oil has been studied thoroughly in the literature [8]. It has a wide variety of medicinal properties, such as analgesic, anesthetic, antiseptic, astringent, carminative, decongestant, expectorant, stimulant, anti-inflammatory, etc. [9,10,11]. Lavender and peppermint oil, like most essential oils, consist of volatile components, which is often a cause of instability during formulation and storage mainly due to evaporation or oxidative degradation [12].

Microencapsulation is a feasible approach to incorporate liquid substances into carriers, thus protecting the essential oils from decomposition or evaporation and improving their stability. Currently, microencapsulation is widely applied in different industrial fields, such as food, textile, cosmetic, chemical, and pharmaceutical [13]. Among the various microencapsulation approaches, spray-drying represents a simple, flexible, rapid, and low-process-cost technique [14,15]. Spray-drying can be used for thermolabile materials due to its short contact time in the dryer [16]. Moreover, a continuous mode of production is allowed with high encapsulation efficiency and enhanced stability of volatiles. In this method, the material for encapsulation is homogenized with the carrier material and the mixture is fed into a spray-dryer where it is atomized with a nozzle into a fine mist. The hot air encounters the droplets, resulting in the rapid evaporation of the solvent, thus dramatically decreasing the droplet temperature and allowing the instantaneous entrapment of the volatile compound [17]. The main variables in spray-drying that are generally optimized are feeding rate, air inlet temperature, and air outlet temperature. Basically, the morphology of the finished product is related to the drying conditions, which, in turn, affect the wall material robustness and ability to retain the encapsulated material. For the spray-drying of essential oils, the development of a stable coarse formulation of essential oils into the wall material solution is of crucial importance [18]. Emulsion stability is related to the droplet size distribution and the rheological properties of the emulsion. Enhanced emulsion stability because of smaller droplet size is a prerequisite for increased encapsulation efficiency during spray-drying with subsequent high total oil content in the formulated particles [19].

A wide variety of wall materials have been exploited for encapsulation of volatile compounds, including carbohydrates, cellulose derivatives, lipids, some proteins, and gums [20,21]. Lavender and peppermint oils have been included in solid particles applying different production techniques using various polymers, such as chitosan, alginate, gelatin, Arabic gum, ethylcellulose, starch, maltodextrin, and albumine [13,22,23,24,25,26]. The choice of a suitable wall material is crucial for the effective encapsulation of the essential oil in the resulting solid particles. The retention capacity of the polymer used is associated mainly with its physical state and its physicochemical characteristics, such as molecular weight, molecular conformation, and chemical functionality [27]. Arabic gum is a heteropolysaccharide with excellent emulsifying and film-forming properties, good ability to produce microsize particles, and low viscosity at high concentration. However, there are studies suggesting that it alone may not be a sufficient wall material for the encapsulation of certain volatiles [26,27,28,29]. Arabic gum capsules may show a limited capacity against oxidation because they act as semipermeable membranes and its porosity to oxygen could be a negative factor for the shelf life of the core material [27]. The addition of a second polymer in order to seal the walls of the resulting solid structures could lead to more efficient encapsulation of the essential oil and its longer retention and stabilization in the final powder product. Maltodextrins are considered as suitable capsule sealing agents, especially for spray-drying. They could reduce the hydroscopicity and enhance the flowability of the resulting material [30].

In our previous research, we have established optimum conditions for the formulation of stable emulsions of lavender and peppermint oils regarding stirring rate, oil-to-water ratio, emulsifier concentration, etc. [31]. In this study, we report the preparation of powder formulations of peppermint and lavender oil using Arabic gum and maltodextrin as wall material for further processing into solid dosage forms for oral administration.

The aim of the study was to evaluate the effect of the wall material type and concentration on the essential oil encapsulation process in order to achieve powder product with a high production yield, maximum oil content in the obtained particles, and satisfactory stability. Moreover, the inclusion of two different essential oils in the proposed particle models allows an assessment to be made of how the oil type affects the characteristics of the final powder product.

## 2. Materials and Methods

### 2.1. Materials

Lavender oil (Ph Eur 9.0), peppermint oil (Ph Eur 9.0), Arabic gum from acacia tree (Ph Eur 9.0), maltodextrin (from maize starch, dextrose equivalent 8.0–15.0), sorbitan monooleate (Span^®^ 80), polyoxyethylene sorbitan monooleate (Tween^®^ 80), and n-hexane were purchased from Sigma-Aldrich, Germany. Analytical standards for gas chromatography analysis (α-pinene, β-pinene, 3-octanon, limonene, linalool, camphor, menthone, menthol, menthyl acetate, caryophyllene) were supplied from Supelco Analytical, Germany.

### 2.2. Determination of Essential Oils Composition by Gas Chromatography (GC)

The components of lavender and peppermint oils were determined using gas chromatograph (GC-CP3800 Varian, UK) equipped with an FID detector, CP 8410 auto sampler, and a VF-5ms capillary column (30 m × 0.25 mm, film thickness 0.25 µm). Nitrogen was used as the carrier gas at a flow rate 1 mL/min. The oven temperature program for both lavender and peppermint oils was initiated at 50 °C and held for 2 min. For peppermint oil, temperature was raised up to 180 °C at a rate 3 °C/min and maintained for 10 min, then raised to 250 °C at a rate 20 °C/min and held 10 min. For lavender oil, temperature was raised to 205 °C at a rate 3 °C/min, maintained 10 min, then raised to 250 °C at a rate 20 °C/min. The injector and detector temperatures were 250 °C and 280 °C, respectively; injection mode split, split ratio 1:100; injected volume 1 µL. The compounds of the oils were identified in terms of their retention times and compared with those of standards; quantification was achieved using peak area calculations.

### 2.3. Emulsion Preparation

Essential oils emulsions were prepared at a final weight of 200 g, containing 2.5% (*w/w*) or 5% (*w/w*) essential oil, 5% (*w/w*) emulsifying blend (Tween^®^ 80 and Span^®^ 80 taken at predetermined amounts in accordance with the critical HLB value of each essential oil), and polymer solution as aqueous phase. Arabic gum and maltodextrin were used as encapsulating materials. Polymer solutions were prepared by adding a certain amount of polymer into purified water to obtain a final solid content of 10% (*w/w*) and 20% (*w/w*). The solutions were stirred overnight at 500 rpm to allow complete hydration of molecules. Emulsification was accomplished by the inversion method under constant stirring (ES mechanical stirrer, Velp Scientifica, Usmate, Italy) at 600 rpm for 20 min. A series of eight emulsions at varied oil concentration and encapsulation polymer ratios was developed.

### 2.4. Spray-Drying

Emulsions were spray dried using a B-290 Büchi Mini Spray Drier (Flawil, Switzerland) equipped with a 0.7 mm diameter nozzle tip hole in open mode. The feed rate and aspirator were set at 7.5 mL/min and 35 m^3^/h, respectively. The temperature at the entrance of the system was 170 °C.

### 2.5. Microparticles Characterization

#### 2.5.1. Production Yield

The production yields of microparticles were calculated using the weight of finally dried microparticles (*W*1) with respect to the initial amounts of the components used (*W*2) using the following equation:

(1) $$Yield\;\left(\%\right)=\frac{W1}{W2}\times 100$$

#### 2.5.2. Particles’ Average Size

The microparticles’ size was determined by dynamic light scattering using a Nanotrac Wave II instrument (Microtrac, York, PA, USA). The system is equipped with 3 mW helium/neon laser at 780 nm wavelength and measures the particle size with noninvasive backscattering technology, performing particle size analysis in the range of 0.8 nm to 6.5 µm. The samples for analysis were prepared by suspending a small amount of the powder particles in isopropyl alcohol as a dispersing medium. All measurements were performed at 20-s intervals and were repeated three times. 

#### 2.5.3. Shape and Surface Morphology

Microstructural attributes of the formulated particles were investigated by scanning electron microscopy using Philips SEM 515 (Philips, Eindhoven, The Netherlands). Samples were analyzed after being attached to the SEM stubs and coated with a thin layer of gold in SC7620 Mini Sputter Coater (Quorum Technologies Ltd., Laughton, East Sussex, UK). SEM was performed at 20 kV acceleration voltage and 5000× magnification.

#### 2.5.4. Surface Oil Content

Surface oil content was determined by dispersing 200 mg microparticles (accurately weighed amount) in 2 mL of hexane for 20 min under slight stirring. Then, the dispersion was centrifuged at 5000 rpm for 5 min, supernatant was filtered through Chromafil^®^ filter (0.45 µm) and analyzed by gas chromatography.

#### 2.5.5. Total Oil Content

The total oil content in the microparticles was determined by hydro-distillation of 10 g of powder in a Clevenger-type apparatus for 3 h at the boiling range of water and atmospheric pressure. The oil volume, read directly from the oil collection arm, was converted to weight by multiplying by its density (0.885 g/mL for lavender oil and 0.898 g/mL for peppermint oil). Determination was carried out in duplicate.

#### 2.5.6. Encapsulation Efficiency

The encapsulation efficiency (*EE*) was calculated as the amount of oil (*O*1) in the total amount of powder with respect to the initial amount of oil used (*O*2) as follows:

(2) $$EE\left(\%\right)=\frac{O1}{O2}\times 100$$

#### 2.5.7. Moisture Content

The moisture content was determined with the loss on drying method using a moisture analyzer Kern MLB 09/2004, (Kern & Sohn GmbH, Balingen, Germany) The sample initial weight was 1 g. Moisture content was expressed as a percentage of weight loss.

### 2.6. Flowability Testing

#### 2.6.1. Angle of Repose (θ)

The angle of repose of the formulated powder samples as an indicator of flowability was measured using fixed funnel method at five different measurements. The angle was calculated according to the equation:

(3) $$\theta =\arctan\frac{h}{r}$$

where θ is the angle of repose, *h*—the height of the cone, *r*—the radius of the cone’s base.

#### 2.6.2. Hausner Ratio (HR)

The Hausner ratio was determined by measuring both the bulk density and the tapped density of the powder. Five grams of microparticle powder were placed into a 25 mL graduated cylinder, the bulk volume V_0_ was measured, and the bulk density (*ρ*_0_) was calculated. The tapped volume V_S_ of the powder was determined by tapping the powder 250 times using SVM tapped density tester (Erweka GmBH, Langen, Germany), and the tapped density (*ρ*_S_) was calculated likewise. The Hausner ratio was found according to the following equation:

(4) $$HR=\frac{\rho_{s}\;}{\rho_{0}\;}$$

### 2.7. X-ray Powder Diffraction (XRPD)

X-ray diffraction studies were carried out using powder X-ray diffractometer (D2 Phaser, Bruker AXS GmbH, Karlsruhe, Germany) to get the idea of the physical state of the constituents. Ni-filtered Cu radiation at 30 kV and 10 mA was used. The scan angle range (2θ) was 4–60°. Powder Diffraction database (ICDD) was used for phase analysis and interpretation of the results.

## 3. Results and Discussion

### 3.1. Characterization of Microparticles

#### 3.1.1. Production Yield and Encapsulation Efficiency

Eight models of oil emulsions were prepared at varied wall polymer concentrations (10% and 20% *w/v*). To investigate the effect of the type of encapsulating agent, two polymers—Arabic gum and maltodextrin—were used as wall materials individually and in combination at different ratios. The essential oils were emulsified in the polymer solutions prior to spray-drying using additional emulsifying agents—Tween 80 and Span 80. The composition of the developed emulsions is presented in Table 1.

The obtained stable emulsions were subjected to spray-drying in a Buchi mini spray drier apparatus under pre-optimized conditions (170 °C inlet temperature, 35 m^3^/h aspiration volume flow, and 7.5 mL/min feed flow). The yield of the resulting microparticles was from 71% to 84%, which could be considered plausible for that preparation method. A slight tendency for increased production yields was observed at higher polymer concentrations, but the effect was insignificant. Using maltodextrin at concentrations above 5% had a slightly greater impact on production yields. Similar results were reported by other research groups, which have also added maltodextrins to reduce the hygroscopicity of the product and thus prevent stickiness and deposition of the material on the wall of the spray-dryer chamber [32,33,34,35]. Maltodextrins are considered to be suitable for spray-drying due to the high solubility, which allows a high degree of incorporation into the feeding solution and, therefore, requires less water removal. The addition of maltodextrin not only improves the drying rate but also enhances powder flowability [30]. There were small differences in the production yields between the samples encapsulating lavender and peppermint essential oils, which were not considered significant.

The estimated encapsulation efficiency of the formulated microparticles was in the range from 19.77% to 91.08% (Table 2). That parameter was significantly influenced by the wall material concentration. By increasing the concentration of the polymer used from 10% to 20%, the encapsulation efficiency increased from 26.53% to 91.08% for the models with lavender oil and from 19.77 to 54.83% for the models with peppermint oil. The higher polymer concentration was required for higher oil retention in the particles and more efficient encapsulation. From the models, formulated with a combination of the two polymers, the microparticles LO-5-AG-MD-15/5 (Arabic gum and maltodextrin in a ratio 3:1) showed the highest encapsulation efficiency of 47.15%.

#### 3.1.2. Particle Shape, Size, and Surface Morphology

The obtained particles were first visualized using an optical microscope (micrographs not shown). Light microscopy revealed that the particles were spherical in shape and tended to aggregate. The average particle diameter was determined using dynamic light scattering, and it varied in the range of 2.41 ± 0.09 to 5.99 ± 0.22 µm (Table 2). The average particle size is generally determined by the process variables, such as drying conditions, method of atomization, concentration, and viscosity of the feeding solution [36].

**Figure 1:**
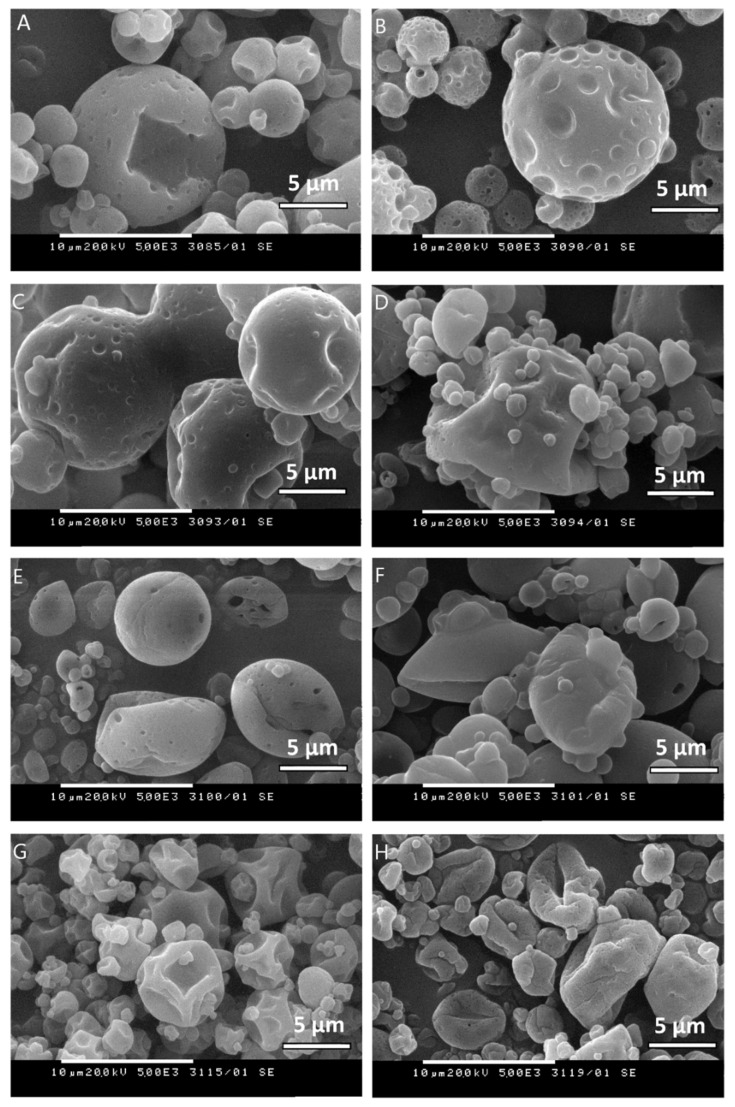
SEM micrographs of the spray dried samples: LO-5-AG-20 (**A**), LO-2.5-AG-10 (**B**), LO-5-AG-MD-15/5 (**C**), LO-5-AG-MD-10/10 (**D**), LO-5-AG-MD-5/15 (**E**), LO-5-MD-20 (**F**), PO-5-AG-20 (**G**), PO-2.5-AG-10 (**H**), magnification 5000×.

The scanning electron microscopy showed that the surface of the different models’ microparticles was heavily grooved with multiple indentations to varying degrees (Figure 1). Numerous pores were found (samples LO-5-AG-20 and LO-2.5-AG-10), with a slight tendency to smoothen the capsule surface when maltodextrin was added to the polymer solution (samples LO-5-AG-MD-15/5, LO-5-AG-MD-10/10, and LO-5-AG-MD-5/15). This gave us the reason to assume that the models containing a combination of the two polymers tend to retain the encapsulated oil in their core for a longer time, which is a prerequisite for achieving a stabilizing effect. These assumptions were later confirmed by a storage stability test. The sample LO-5-MD-20 that contained maltodextrin as the only encapsulating polymer revealed a much smoother surface without pores and cracks. Therefore, particle morphology is mainly governed by the concentration of maltodextrin in the feeding emulsion. The mixing of Arabic gum with maltodextrin resulted in favorable particle characteristics probably due to the decreased viscosity of the feeding emulsion. Generally, an ideal wall material should have an optimum film forming property and emulsifying ability. The low viscosity of the feeding liquid is essential for the emulsion stability and evaporation rate, ensuring the rapid solidification of the droplets and obtaining smoother particle surface. In the samples with encapsulated peppermint oil (samples PO-5-AG-20 and PO-2.5-AG-10), there were serious deformations in the shape of the particles, which were much more pronounced in the sample PO-2.5-AG-10. The microparticles obtained at a lower polymer concentration (10%) presented rough and uneven surfaces, suggesting that the wall is unable to prevent the penetration of the encapsulated oil and assure nucleus protection. From the obtained result, we could suppose that Arabic gum is not capable of forming continuous stable film around the oil droplets before the drying process. This correlates with the unsatisfactory results in terms of the content of essential oil in the polymer structures.

### 3.2. XRD Analysis

**Figure 2:**
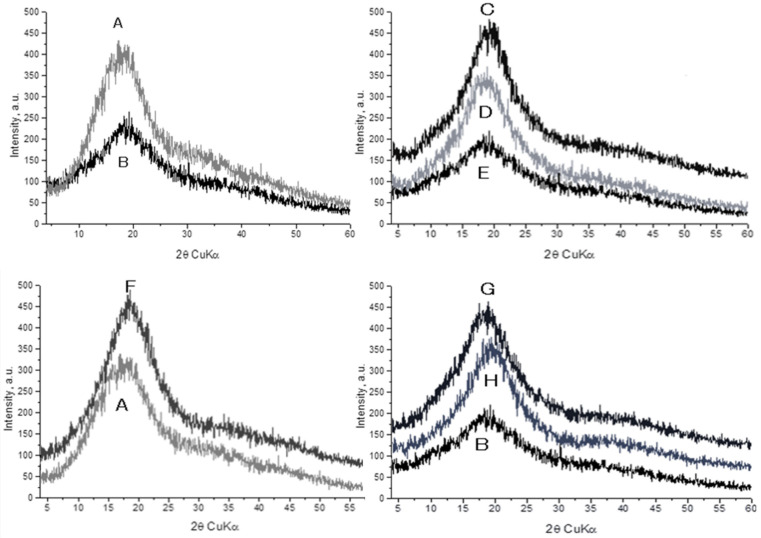
XRD spectra of the raw materials and the formulated samples: maltodextrin (**A**), Arabic gum (**B**), LO-2.5-AG-10 (**C**), LO-5-AG-20 (**D**), LO-5-AG-MD-10/10 (**E**), LO-5-MD-20 (**F**), PO-5-AG-20 (**G**), PO-2.5-AG-10 (**H**).

The X-ray diffraction results indicated that the controls (Arabic gum and maltodextrin) and the samples (microparticles of lavender and peppermint oils) had similar profiles, characteristic of an amorphous structure. No diffraction maxima of crystalline phases were found in the samples (Figure 2). An intense broad peak was recorded in all the samples, probably due to the presence of coherently scattering domains (structural blocks). The diffraction patterns of the samples were similar, suggesting that the type of the encapsulated oil and the wall materials ratio did not affect the structure of the developed microparticles.

### 3.3. Total Oil Content

The percentage of total oil in the formulated polymer microparticles varied from 1.34% to 10.80% (Table 2), indicating that the evaluated parameter was significantly affected by the wall material type (Arabic gum, maltodextrin, or their combination) and its concentration (10% or 20%). Higher oil retention values were observed at high content of solids (20%) compared to samples prepared at 10% wall material concentration. A tendency to reduce the amount of essential oil encapsulated in the particles was observed when a lower concentration of wall polymers was used. A polymer concentration of 10% was insufficient to effectively retain the essential oil in the particles, probably due to the evaporation of the oil during the spray-drying process. The concentration of wall materials in the feeding solution for spray-drying is directly related to the viscosity of the medium, which, in turn, affects the total oil content retained in the particles. Numerous studies suggested that there should be optimum concentrations of each wall material to achieve the maximum retention of volatile substances at optimized viscosity of the solution [37,38]. The formation of discrete particles is hindered when the feeding solution viscosity is too high, which can result in significant losses of volatile components during atomization. On the other hand, low viscous solutions would require a longer time to form a semi-permeable barrier at the beginning of the drying process so that evaporation of essential oils is prevented [22,39]. Furthermore, the total oil content was significantly affected by the type of the encapsulating oil—lavender or peppermint oil. Dramatic differences were observed in samples prepared under the same conditions, varying only the type of the encapsulated oils, especially at a low polymer concentration of 10%. The total content of lavender oil was 5.31%, whereas the peppermint oil retained in the particles was only 1.34%. This could be attributed to the formation of particles with multiple invaginations and indentations, and small volume capacity for oil encapsulation, as seen from the scanning electron microscopy (Figure 1, sample PO-2.5-AG-10). This was confirmed by encapsulation efficiency data (19.77%) suggesting that a great amount of peppermint oil was lost during atomization due to evaporation. The highest encapsulation efficiency (91.08%) was observed at sample LO-5-MD-20, prepared using maltodextrin and lavender oil, proving the hypothesis that the optimum combination of feeding material and drying conditions is needed for achieving the maximum encapsulation efficiency and oil retention.

### 3.4. Surface Content of Essential Oils in the Particles

The surface oil content of essential oil microparticles is often used as an important tool for assessing their stability [23,40]. In addition, powders with high surface oil content are prone to sticking and cohesion, leading to poor flowability [37]. The surface oil contents of the formulated microparticle models ranged from 0.93% to 2.07%. The registered low surface oil content is also an indicator of the efficient encapsulation of the oil inside the obtained polymer particles.

#### 3.4.1. Surface Content of Lavender Oil

The particle surface oil content was evaluated using gas chromatographic analysis after extraction of the oil from the surface of the microparticles with hexane for 15 min. The lavender oil components content (α-pinene, β-pinene, 3-octanone, cineole, linalool, camphor, lavender, terpinene-4-ol, menthyl acetate, caryophyllene) was determined. The surface oil content was calculated based on linalool and camphor, which predominate in the composition of lavender oil. The results are presented in Table 3. The surface content in all the samples is relatively low (ranging from 1.13% to 2.07%) compared to the total content of essential oil in the microparticles, which is a reason to believe that high microencapsulation efficiency has been achieved. The variations between the individual models can be explained by the influence of maltodextrin in some of the compositions as a capsule sealant. With an increase in the amount of maltodextrin, there is a tendency to decrease the surface oil content and priority distribution of the oil in the core of the microparticles.

#### 3.4.2. Surface Content of Peppermint Oil

Particle surface oil content was evaluated using gas chromatographic analysis after extraction of the oil from the surface of the microparticles with hexane for 15 min. The quantitative content of the components of the oil (α-pinene, β-pinene, 3-octanone, limonen, linalool, camphor, menthone, menthol, menthyl acetate, caryophyllene) was determined. The surface content of peppermint oil in the microparticles was calculated based on menthol, which predominated in the composition of peppermint oil. The obtained results are presented in Table 4.

According to the obtained results, the surface oil content was relatively low (around 1.5% from the total oil content). An insignificant difference between the two evaluated sample models was observed.

### 3.5. Moisture Content

The moisture content of the microparticles (Table 5) ranged from 3.65 to 5.51%. The values obtained were similar to those reported by other research groups working on spray-drying of essential oils: 1.70—4.16%; 1.20—2.70% [24,41].

The effect of process variables on particle moisture has been thoroughly studied and verified by numerous researchers. The main spray-drying parameters that influence the product moisture content are the inlet temperature, air-drying temperature, and feed rate [42]. In the present study, we examined the effect of the wall material type and concentration. The residual moisture in the formulated powders increased when a higher concentration of Arabic gum was used. This may be due to the large-sized molecules of the polymer, which prevent water diffusion during spray-drying. In addition, higher amounts of encapsulating material are usually associated with a shorter time to form the capsule shell formation, which also contributes to hindered water diffusion [43]. Furthermore, there was a clear trend towards reduced moisture content when the concentration of maltodextrin was increased. That was also observed in a study by Fasaeli et al. and could be explained by the fact that maltodextrins with low dextrose equivalent (DE = 10–15), as the type used for the present work, are less hygroscopic than higher DE maltodextrins and have a lower tendency to water retention [44].

### 3.6. Flowability Study

Powder flow properties are essential for the further processing of the prepared microparticles into solid dosage forms (tablets or capsules). The flowability of the formulated particle models was investigated by determining the angle of repose and the Hausner ratio. The angle of repose (Table 5) was greater than 35°, indicating fair to passable flow properties [45]. A gradual decrease in the angle of repose was noticed with the addition of maltodextrin as a wall material. Moreover, the higher the maltodextrin concentration, the smaller the angle of repose. The smallest angle of repose (39°) related to satisfactory flowability was achieved in the sample containing maltodextrin as the only encapsulating polymer. Probably, that was due to the lower moisture content of the microparticles. On the other hand, the lower surface oil content of the models increased the stickiness between the microparticles, which probably led to a deterioration of the flowability.

## 4. Conclusions

In this study, lavender and peppermint essential oils were successfully encapsulated by emulsification with Arabic gum, Tween 80 and Span 80, and a subsequent spray-drying process at 170 °C temperature, 35 m^3^/h aspiration flow, and 7.5 mL/min feed flow. The particles obtained had irregular surfaces and mean diameters from 2.41 to 5.99 µm. The process parameters that mainly affected the yields, particle size, and oil content were the type and concentration of the wall material. A polymer concentration of 20% was required for higher oil retention in the particles. The results show that Arabic gum alone could not form a continuous and stable film around the oil droplets. The addition of maltodextrin as a second polymer proved to be a successful approach for further sealing the walls of the resulting solid structures and improving their morphological characteristics. From the varied ratios between Arabic gum and maltodextrin, 3:1 could be outlined as optimal, resulting in particles with high encapsulation efficiency and a high total essential oil content of 10.55%. Our study has demonstrated that the encapsulation of lavender and peppermint oils in Arabic gum/maltodextrin microparticles by spray-drying represents a feasible approach for the conversion of liquid materials into solids with satisfactory flowability for further use in powder technology.

## Figures and Tables

**Table 1:** Experimental design of emulsions for spray-drying.

Sample Code	Lavender Oil (LO)		Peppermint Oil (PO)		Arabic Gum (AG)		Maltodextrin (MD)		Tween 80	Span 80
	g	%	g	%	g	%	g	%	g	g
LO-5-AG-20	10	5	-	-	40	20	-	-	5.33	4.67
LO-2.5-AG-10	5	2.5	-	-	20	10	-	-	5.33	4.67
LO-5-AG-MD-15/5	10	5	-	-	30	15	10	5	5.33	4.67
LO-5-AG-MD-10/10	10	5	-	-	20	10	20	10	5.33	4.67
LO-5-AG-MD-5/15	10	5	-	-	10	5	30	15	5.33	4.67
LO-5-MD-20	10	5	-	-	-	-	40	20	5.33	4.67
PO-5-AG-20	-	-	10	5	40	20	-	-	7.48	2.52
PO-2.5-AG-10	-	-	5	2.5	20	10	-	-	7.48	2.52

**Table 2:** Characterization of the microparticles prepared at varied wall material type and concentration (LO = lavender oil, PO = peppermint oil, AG = Arabic gum, MD = maltodextrin; *n* = 3).

Sample Code	Wall Material	Wall Material Concentration, %	Production Yield, % ± SD	Mean Particle Size, µm ± SD	Total Oil Content, % ± SD	Encapsulation Efficiency %± SD
LO-5-AG-20	AG	20	76.45 ± 2.03	5.93 ± 0.18	10.62 ± 0.72	48.72 ± 0.53
LO-2.5-AG-10	AG	10	71.37 ± 1.87	3.15 ± 0.12	5.31 ± 0.91	26.53 ± 0.95
LO-5-AG-MD-15/5	AG/MD 75/25	20	74.01 ± 0.98	4.84 ± 0.14	10.55 ± 1.12	47.15 ± 1.11
LO-5-AG-MD-10/10	AG/MD 50/50	20	83.05 ± 1.23	5.99 ± 0.22	8.85 ± 0.57	44.10 ± 1.02
LO-5-AG-MD-5/15	AG/MD 25/75	20	81.03 ± 1.92	2.41 ± 0.09	7.08 ± 1.02	34.42 ± 0.97
LO-5-MD-20	MD	20	84.33 ± 0.85	3.84 ± 0.21	10.80 ± 1.71	91.08 ± 1.78
PO-5-AG-20	AG	20	76.37 ± 1.48	3.32 ± 0.08	7.18 ± 0.65	54.83 ± 1.53
PO-2.5-AG-10	AG	10	73.77 ± 0.97	3.15 ± 0.07	1.34 ± 0.46	19.77 ± 0.61

**Table 3:** Surface content and total amount of lavender oil in microparticles.

Sample Code	Surface Content of LO in Microparticles Calculated as Percentage of:		Total Amount of LO, %	Surface Content of LO Relative to the Total Amount, %
	Linalool, %	Camphor, %		
LO-5-AG-20	0.21	0.22	10.62	2.07
LO-2.5-AG-10	0.11	0.08	5.31	1.51
LO-5-AG-MD-15/5	0.22	0.20	10.55	1.90
LO-5-AG-MD-10/10	0.10	0.10	8.85	1.13
LO-5-AG-MD-5/15	0.10	0.10	7.08	1.41
LO-5-MD-20	0.10	0.10	10.80	0.93

**Table 4:** Surface content and total amount of peppermint oil in microparticles.

Sample Code	Surface Content of PO in Microparticles Calculated as Percentage of: Menthol, %	Total Amount of PO, %	Surface Content of PO Relative to the Total Amount, %
PO-5-AG-20	0.11	7.18	1.53
PO-2.5-AG-10	0.02	1.34	1.49

**Table 5:** Moisture content and flow properties of the developed microparticles (*n* = 3).

Sample Code	Moisture Content, % ± SD	Angle of Repose, ° ± SD	Hausner Ratio ± SD
LO-5-AG-20	5.51 ± 0.23	49.48 ± 0.24	1.875 ± 0.03
LO-2.5-AG-10	4.39 ± 0.10	53.57 ± 0.51	1.852 ± 0.05
LO-5-AG-MD-15/5	4.05 ± 0.06	50.16 ± 0.68	1.816 ± 0.08
LO-5-AG-MD-10/10	3.74 ± 0.12	45.78 ± 1.08	1.592 ± 0.06
LO-5-AG-MD-5/15	3.65 ± 0.14	43.55 ± 0.40	1.518 ± 0.10
LO-5-MD-20	3.58 ± 0.03	39.00 ± 0.99	1.432 ± 0.07
PO-5-AG-20	4.78 ± 0.18	46.61 ± 1.13	1.687 ± 0.02
PO-2.5-AG-10	4.36 ± 0.21	44.96 ± 1.37	1.514 ± 0.01

## Data Availability

The data presented in this study are available on request from the corresponding author.

## Sample Availability

Samples of the compounds are available from the authors.
